# A novel FRET pair for detection of parallel DNA triplexes by the LightCycler

**DOI:** 10.1186/1472-6750-10-4

**Published:** 2010-01-27

**Authors:** Uffe V Schneider, Jette K Severinsen, Imrich Géci, Limei M Okkels, Nina Jøhnk, Nikolaj D Mikkelsen, Teena Klinge, Erik B Pedersen, Henrik Westh, Gorm Lisby

**Affiliations:** 1 QuantiBact Inc, Department of Clinical Microbiology, Hvidovre Hospital, Kettegaards Alle 30, 2650 Hvidovre, Denmark; 2 Nucleic Acid Center and Department of Chemistry, University of Southern Denmark, Campusvej 55, 5230 Odense M, Denmark; 3 Department of Clinical Microbiology, Hvidovre Hospital, Kettegaards Alle 30, 2650 Hvidovre, Denmark; 4 Faculty of Health, University of Copenhagen, Copenhagen, Denmark

## Abstract

**Background:**

Melting temperature of DNA structures can be determined on the LightCycler using quenching of FAM. This method is very suitable for pH independent melting point (Tm) determination performed at basic or neutral pH, as a high throughput alternative to UV absorbance measurements. At acidic pH quenching of FAM is not very suitable, since the fluorescence of FAM is strongly pH dependent and drops with acidic pH.

Hoogsteen based parallel triplex helix formation requires protonation of cytosines in the triplex forming strand. Therefore, nucleic acid triplexes show strong pH dependence and are stable only at acidic pH. This led us to establish a new pH independent fluorophore based measuring system on the LightCycler for thermal stability studies of parallel triplexes.

**Results:**

A novel LightCycler FRET pair labelled with ATTO495 and ATTO647N was established for parallel triplex detection with antiparallel duplex as a control for the general applicability of these fluorophores for Tm determination. The ATTO fluorophores were pH stable from pH 4.5 to 7.5. Melting of triplex and duplex structures were accompanied by a large decrease in fluorescence intensity leading to well defined Tm and high reproducibility. Validation of Tm showed low intra- and inter-assay coefficient of variation; 0.11% and 0.14% for parallel triplex and 0.19% and 0.12% for antiparallel duplex. Measurements of Tm and fluorescence intensity over time and multiple runs showed great time and light stability of the ATTO fluorophores. The variance on Tm determinations was significant lower on the LightCycler platform compared to UV absorbance measurements, which enable discrimination of DNA structures with very similar Tm. Labelling of DNA probes with ATTO fluorophore increased Tm of antiparallel duplexes significantly, but not Tm of parallel triplexes.

**Conclusions:**

We have established a novel pH independent FRET pair with high fluorescence signals on the LightCycler platform for both antiparallel duplex and parallel triplex formation. The method has been thoroughly validated, and is characterized by an excellent accuracy and reproducibility. This FRET pair is especially suitable for ΔTm and Tm determinations of pH dependent parallel triplex formation.

## Background

**Figure 1:**
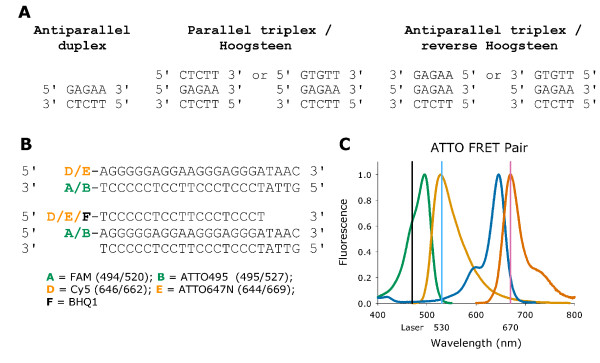
**Illustration of triplex DNA structures and FRET pairs**. (A) Randomly choosen polypurine sequence illustrating the orientation and sequence of antiparallel duplex, Hoogsteen type parallel triplexes and reverse Hoogsteen type antiparallel triplexes. (B) FRET pairs used for Watson-Crick antiparallel duplex formation and Hoogsteen parallel triplex formation with fluorophore name and brackets with excitation and emission maximum (nm). (C) Graphic presentation of the ATTO fluorophore FRET pair. Excitation and emission curves are shown for ATTO495 (left) and ATTO647N (right). Vertical lines demonstrate LightCycler laser excitation at 470 nm and detection channels at 530 and 670 nm. The graph is generated from spectral data obtained from http://www.ATTO-tec.com. Data was recorded by UV absorbance.

Triplex-forming oligonucleotides (TFO) have attracted considerable interest due to their potential as therapeutics for gene targeting, which allows transcriptional control, gene knock-out and sequence-selective treatment [1,2]. Furthermore, TFO can be used for recognition and purification of DNA [3,4]. TFO bind in the major groove on homopurine sequences of Watson-Crick-based antiparallel duplex DNA and are divided into Hoogsteen and reverse Hoogsteen formations by orientation of the third strand [5,6]. Hoogsteen formation is based upon the parallel binding of a TFO consisting of CT or GT sequences to the homopurine sequence of the antiparallel duplex DNA [7], whereas reverse Hoogsteen is formed by a TFO consisting of GT or GA sequences in antiparallel binding with the homopurine sequence of the antiparallel duplex DNA (e.g. in Figure 1a) [5]. Antiparallel duplex -, reverse Hoogsteen- and GT Hoogsteen-formations are pH independent, whereas the formation of Hoogsteen CT parallel triplexes are pH dependent due to the need for protonated cytosine in the TFO [7-9].

Melting points (Tm) for DNA duplex and triplex are often investigated by UV absorbance based on melting curve analysis, and this method is widely used for evaluation of novel artificial nucleic acid analogs [10-12]. Although UV absorbance is a commonly used method and considered as the golden standard, it is limited by: 1) a relatively low sample throughput; 2) the need for relatively large amounts of oligonucleotides; 3) a relatively low switch in absorbance level upon melting; and 4) the possibility for overlapping peaks for each strand composition in the melting profile [13]. As an alternative to UV absorbance, melting curve analysis can be performed using real-time polymerase chain reaction (PCR) platforms e.g. the LightCycler [13]. Real-time PCR platforms are available in many laboratories and melting curves are already used routinely for evaluation of PCR products and even as a tool for genotyping [14,15].

In 2002 a LightCycler based method for melting point determination of duplex, triplex and quadruplex formation using oligonucleotides labelled with fluorescein (FAM) and a quencher (methyl red) was published [13]. The fluorescence intensity of FAM is well known to be highly pH dependent and to decrease towards acidic pH. As a consequence, the melting curve is very flat and the change in fluorescence is only 1-3 LightCycler units on a 100 units scale at acidic pH [13,16,17]. Such small changes in fluorescence are likely to influence the accuracy of Tm and ΔTm and a new pH stable fluorophore pair is needed for detection of pH-dependent parallel triplexes. ATTO fluorophores could be used as such a fluorophore pair and has previously been applied to melting point determinations of antiparallel duplexes at neutral pH [18]. We describe a novel pH-independent fluorescence resonance energy transfer (FRET)-based ATTO fluorophore pair using non-linked oligonucleotides to study the thermal stability of Watson-Crick-based DNA antiparallel duplex and Hoogsteen-based DNA parallel triplex. Furthermore, we have thoroughly validated our method and compared it to UV absorbance measurements.

## Results

### Evaluation of FRET pairs

**Figure 2:**
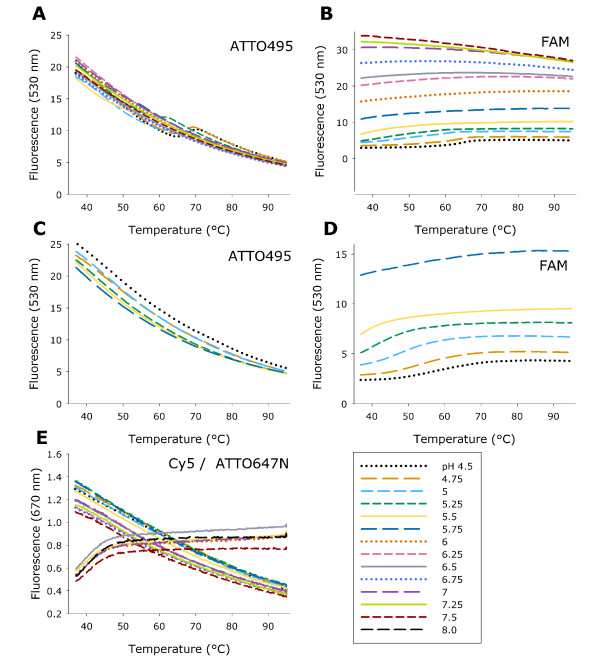
**Melting curves demonstrating pH and temperature dependence of ATTO, FAM and Cy5 fluorophores**. (A, B) Fluorescence of ATTO495 and FAM at 530 nm from pH 4.5 to 7.5 (1 μM probe) in cacodylate buffer with MgCl_2_. (C, D) Fluorescence of ATTO495 and FAM at 530 nm from pH 4.5 to 5.75 in cacodylate buffer without MgCl_2_. (E) Fluorescence of Cy5 (decreasing by temperature) and ATTO647N (increasing by temperature) at 670 nm from pH 4.5 to 7.5 in sodium cacodylate buffer (1 μM probe).

To develop a pH independent FRET system, two comparable FRET-pairs consisting of ATTO495-ATTO647N and FAM-Cy5 were constructed (Figure 1b, c). Each fluorophore was evaluated independently for temperature and pH dependence in sodium cacodylate buffers (Figure 2a, b). For ATTO495, the fluorescence was independent of pH, but decreased with increasing temperature (Figure 2a). When the temperature was between 50°C and 70°C minor variations in fluorescence were observed at acidic pH; such variations were eliminated when magnesium chloride was excluded from the buffer (Figure 2c). The background fluorescence of FAM changed significantly with both pH and temperature (Figure 2b). When pH increased, the fluorescence intensity of FAM increased. At acidic pH, an increase in fluorescence with increasing temperature was observed, whereas the opposite was observed at neutral pH. Only minor changes in fluorescence of FAM with temperature were found in buffers without magnesium chloride (Figure 2d). ATTO647N and Cy5 were only slightly excitated by the laser resulting in low background fluorescence. For ATTO647N and Cy5 no pH dependence was observed (Figure 2e).

#### Antiparallel duplex

**Figure 3:**
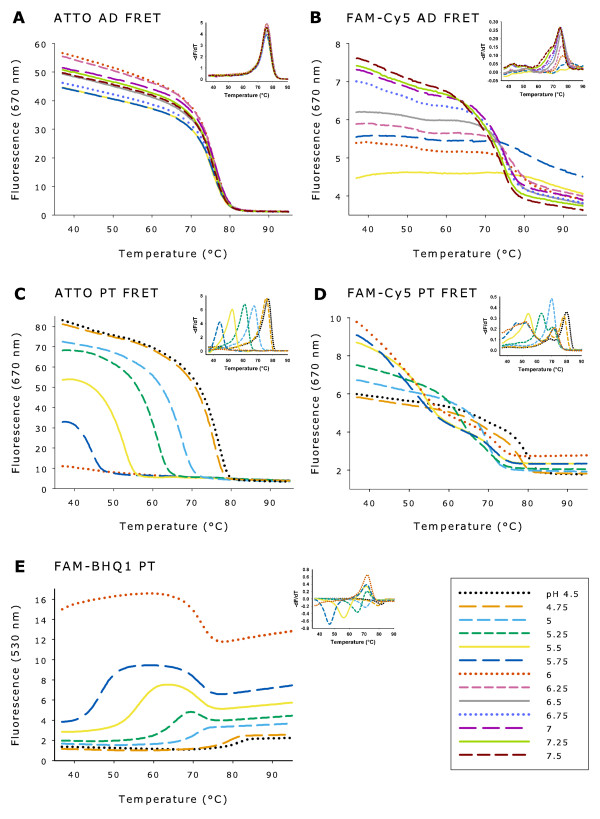
**Melting curves demonstrating pH dependence of antiparallel duplex and parallel triplex FRET-pairs**. All experiments in sodium cacodylate buffer with MgCl_2_. (A, B) Fluorescence of ATTO495-647N and FAM-Cy5 antiparallel duplex (AD) FRET pairs at 670 nm from pH 5.5 to 7.5 (0.5 μM of each probe). (C, D) Fluorescence of ATTO495-ATTO647N and FAM-Cy5 parallel triplex (PT) FRET pairs at 670 nm from pH 4.5 to 6.0 (1.0 μM of each probe). (E) Fluorescence of FAM-BHQ1 parallel triplex at 530 nm from pH 4.5 to 6.0 with 1.0 μM of each probe.

The ATTO495-ATTO647N FRET pair designed for Tm determinations of antiparallel duplex formation was pH independent from pH 5.5 to 7.5 (Figure 3a), whereas the fluorescence level of the FAM-Cy5 FRET pair changed greatly with pH (Figure 3b). At pH below 6.0, no FAM-Cy5 melting curves could be defined. Moreover, the decrease in the FRET-based fluorescence was larger in the ATTO495-ATTO647N system (>96%) compared to the FAM-Cy5 system (<44%), indicating a more accurate Tm determination in the ATTO system (Figure 3a, b).

#### Parallel triplex

As seen in Figure 3c and 3d, Tm for parallel triplex changed (parallel shifts) with pH for both FRET pairs. The fluorescence of ATTO495-ATTO647N triplex formation decreased at increasing pH values, clearly depicting the decreased stability of triplex formation from pH 4.5 to 5.75, but even at pH 5.75 a well-defined melting peak was observed - in contrast to the FAM-Cy5 triplex formation, where no melting peak could be determined at pH 5.75. For ATTO495-ATTO647N, a large decrease in the level of fluorescence (>77%) was observed upon melting throughout the acidic pH-range (4.5 - 5.75). The melting curves for FAM-Cy5 were difficult to interpret, even in the optimal 'triplex forming' pH range (pH 4.5 - 5.5). This is due to the low fluorescence level and small decrease (<29%) upon melting. Similar results were obtained for FAM-Black Hole Quencher 1 (BHQ1). In this system only a modest increase in fluorescence (<121% of background fluorescence) was observed upon melting (Figure 3e).

#### Buffer types and probe concentrations

**Figure 4:**
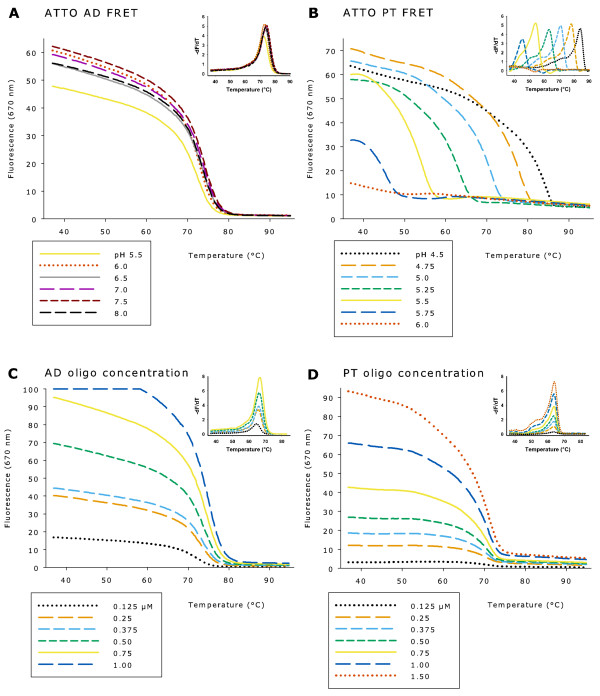
**Melting curves after change of buffer and effect on Tm of probe concentrations**. (A) Fluorescence of ATTO495-ATTO647N antiparallel duplex (AD) FRET at 670 nm from pH 5.5 to 8.0 with 0.5 μM of each probe in sodium phosphate buffer. (B) Fluorescence of ATTO495-ATTO647N parallel triplex (PT) FRET at 670 nm from pH 4.5 to 6.0 with 1.0 μM of each probe in sodium acetate buffer. (C) ATTO495-ATTO647N antiparallel duplex FRET at 670 nm in sodium phosphate buffer pH 7.0 and probe concentrations from 0.125 μM to 1.00 μM of each probe. (D) ATTO495-ATTO647N parallel triplex FRET at 670 nm in sodium acetate buffer pH 5.0 and probe concentrations from 0.125 μM to 1.50 μM of each probe.

The Tm of ATTO495-647N antiparallel duplex formation was lower in sodium phosphate buffer compared with sodium cacodylate buffer, whereas the opposite was the case for parallel triplex formation in sodium acetate buffer compared with sodium cacodylate buffer (Figure 3a, c and Figure 4a, b).

Different concentrations of ATTO495-647N probes from 0.125 μM to 1.50 μM were found to change the level of fluorescence, but not the Tm determination for antiparallel duplex and parallel triplex formation (Figure 4c, d).

### LightCycler program validation

**Figure 5:**
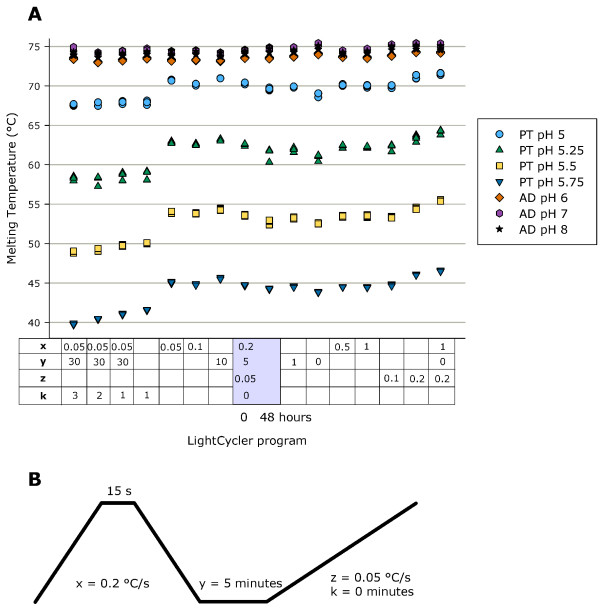
**LightCycler program and effect of program changes**. (A) Quadruplicate runs of antiparallel duplex formation at pH 6, 7 and 8 in sodium phosphate buffers with 0.5 μM of each probe and parallel triplex formation at pH 5, 5.25, 5.5 and 5.75 in sodium acetate buffers with 1.0 μM of each probe. Using the same capillaries for 16 runs. The first run and last run were performed at standard conditions. Empty boxes are standard conditions as written in the grey box. AD is antiparallel duplex and PT is parallel triplex. (B) LightCycler standard program. x = dissociation and annealing speed (°C/second), y = hold time (minutes), z = dissociation speed before measurement (°C/second), k = hold before each measurement (minutes).

To ensure the robustness of the melting curve determination on the LightCycler platform when using the ATTO495-ATTO647N FRET pair, different variations of the LightCycler program were performed (Figure 5a, b). Changes in all program steps except the final dissociation step (sample annealing, dissociation and time at constant temperature) had minor influence on Tm. With increasing melting curve dissociation speed, Tm for parallel triplex increased, whereas dissociation time had no effect on Tm of antiparallel duplex (Figure 5a).

### Validation of the LightCycler system

**Figure 6:**
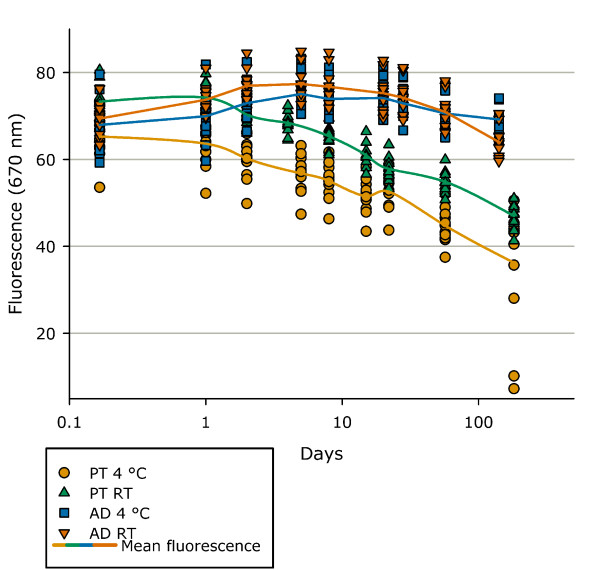
**Fluorescence of prepared probe mixtures over time and multiple runs**. Antiparallel duplex stability in sodium phosphate buffer, pH 7.0 kept at 4°C (AD 4°C) or room temperature (AD RT) and parallel triplex stability in sodium acetate buffer, pH 4.8 kept at 4°C (PT 4°C) or room temperature (PT RT). Fluorescence is measured at 37°C.

When antiparallel duplex Tm determinations were run on one LightCycler, the intra-assay coefficient of variation was 0.19%, whereas the inter-assay coefficient of variation was 0.12% (Table 1). When running the assay simultaneously on two LightCyclers, the inter-machine variation for antiparallel duplex formation Tm determination was 0.17°C (CI 0.13; 0.22). This was due to a parallel shift in Tm and not to random variation. Probes and buffer could be mixed and kept at 4°C before the LightCycler run for two days without any significant changes in Tm. The ATTO fluorophores were very stable and the mean fluorescence level did not change for capillaries left at 4°C or room temperature for 141 days (Figure 6).

**Table 1:** Validation of Tm determinations for antiparallel duplex and parallel triplex formations

	Mean (°C)	Standard Deviation	Number	Minimum (°C)	Maximum (°C)	CV%
**Antiparallel duplex**						
Intra assay	73.60	0.14	12	73.33	73.80	0.19
Inter assay	73.65	0.09	6	73.52	73.80	0.12
**Parallel triplex**						
Intra assay	75.03	0.08	12	74.89	75.13	0.11
Inter assay	75.11	0.10	6	75.01	75.25	0.14

Intra- and inter-assay coefficient of variation for antiparallel duplex formation in sodium phosphate buffer pH 7.0 with 0.5 μM of each probe and parallel triplex formation in sodium acetate buffer pH 4.8 with 1.0 μM of each probe.

For parallel triplex formation the intra-assay coefficient of variation on Tm determination was 0.11%, and the inter-assay coefficient of variation on this LightCycler was 0.14% (Table 1). When using two LightCyclers a parallel shift in Tm of 0.09°C (CI 0.07; 0.10) was found. Mixed probes and buffer could be kept at 4°C for eight days before the LightCycler run without any significant changes in Tm. The mean fluorescence level of the ATTO fluorophores for parallel triplex formation (measured at 37°C) was found to decrease for capillaries left at 4°C or room temperature. After 57 days, a mean fluorescence level of 44.77 for capillaries left at 4°C and 54.87 for capillaries left at room temperature compared to initially 69.26/69.93 was found (Figure 6).

### Comparison of UV-absorbance and LightCycler Tm determination

**Figure 7:**
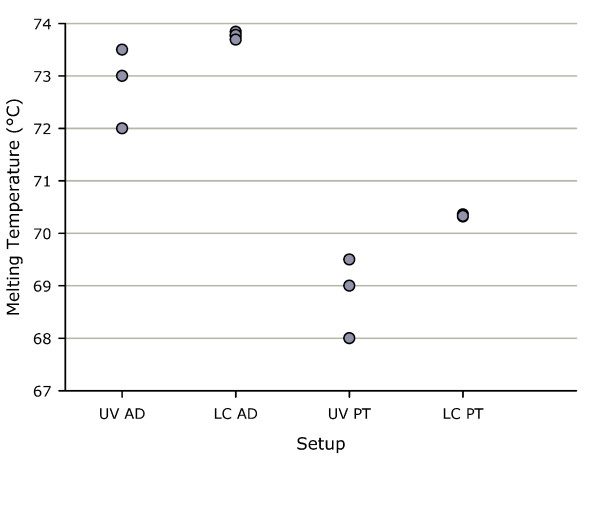
**Tm determined by UV absorbance compared with Tm on the LightCycler**. Quadruplicate determinations for antiparallel duplex formation by UV absorbance (UV AD) as 1.0 μM of each probe and LightCycler (LC AD) in sodium phosphate buffer pH 7.0 as 0.5 μM of each probe and for parallel triplex formation by UV absorbance (UV PT) and LightCycler (LC PT) in sodium acetate buffer pH 5.0 with 1.0 μM of each probe.

For antiparallel duplex formation, the mean Tm of quadruplicate measurements by UV-absorbance was 72.88°C with a standard deviation (std dev) of 0.63°C and a range from 72.00 to 73.50°C, whereas the mean Tm determined by LightCycler was 73.77°C with a std dev of 0.06°C and a range from 73.69 to 73.84°C (Figure 7). UV absorbance measurements of probe mixtures without ATTO fluorophores showed a mean Tm of 67.25°C with a std dev of 0.65°C and range from 66.50 to 68.00°C. The variance of Tm by UV absorbance was 0.40 compared to 0.004 on the LightCycler (p = 0.003).

For parallel triplex formation UV-absorbance measurements of Tm was 69.00°C with a std dev of 0.71°C and a range from 68.00 to 69.50°C, compared to a Tm of 70.34°C with a std dev of 0.02°C and a range from 70.32 to 70.36°C by LightCycler determination (Figure 7). Parallel triplex measurements without ATTO fluorophores by UV absorbance had a mean Tm of 68.25°C with a std dev of 0.50°C and a range from 67.50 to 68.50°C. The variance of Tm was 0.50 by UV absorbance compared with 0.0003 on the LightCycler (p < 0.0001).

## Discussion

Fluorescein is unionised at acidic pH and the fluorescence intensity changes with pH [19,20]. As expected, our results clearly demonstrate this effect on fluorescence levels using a FRET pair and a quencher pair with FAM. By use of the ATTO495-ATTO647N FRET pair for Hoogsteen-based parallel triplex formation, a robust and reliably LightCycler method was established. This novel FRET pair is well-suited for Tm and ΔTm determinations over a broad pH range of parallel triplex formations.

Furthermore, this FRET pair clearly demonstrates the pH independence from pH 5.5 to 7.5 of antiparallel duplex Tm determinations in contrast to the pH dependent Tm determination of parallel triplex formation from pH 4.5 to 6.0. An interesting feature of this is the negative correlation between pH and fluorescence intensity as pH increases from 4.5 to 6.0 for parallel triplex formation (Figure 3c). This is in concordance with the expected lower efficacy of parallel triplex formation due to the lack of protonated cytosine at less acidic pH [7]. This effect could not be demonstrated with FAM-based FRET, because the intensity of the FAM fluorescence increases with pH.

Changing the buffer system lead to alterations in Tm determination, especially for parallel triplex formation in sodium acetate compared with sodium cacodylate buffers. The melting point changes might be explained by different stability of parallel triplex formation in different buffers and different monovalent cation concentrations [21,22].

Overall, pH, buffer and LightCycler program were found to influence the melting point determination for parallel triplex formation. The reasons, why we validated our method to a dissociation ramp rate of 3°C/min instead of 1°C/min, as generally used for Tm determinations by UV absorbance, were: 1) that changes in the LightCycler program led to uniform parallel shifts of Tm independent of pH; 2) an extremely low inter-assay variation despite the faster dissociation rate; 3) the LightCycler program was shortened with almost one hour. As long as the LightCycler program is described and it is not changed within a study, we recommend the faster dissociation rate.

In the current study melting temperatures determined by LightCycler were consistently higher than those obtained by UV-absorbance and reflect the higher dissociation ramp rate chosen for LightCycler Tm determinations. The variance of individual melting point measurements by LightCycler was significantly reduced compared to UV absorbance measurements. The high reproducibility of LightCycler determinations is especially important when small melting point differences are to be determined.

The presence of ATTO fluorophores increased Tm of antiparallel duplex formation measured by UV absorbance. This was surprising, but modifications such as quenchers have previously been reported to alter Tm up to 4°C [23]. The presence or absence of ATTO fluorophores did not alter Tm of parallel triplex formation determined by UV absorbance, which suggest a steric proximity of the ATTO fluorophores in antiparallel duplex formation compared with parallel triplex formation.

## Conclusions

Based upon a novel pair of pH stable ATTO fluorophores, a pH stable FRET system was established on the LightCycler platform. This system is highly suitable for melting point determination of Watson-Crick duplex formation and pH dependent Hoogsteen-based parallel triplex formation. The method has been thoroughly validated and we have shown that the variance on melting point determinations is significantly smaller when measured by the LightCycler compared to measurements by UV absorbance. This high throughput and low cost method can be used to measure and distinguish even very small differences in Tm and ΔTm in a variety of applications within molecular biology.

## Methods

### Oligonucleotides

A 19 nucleotide triplex TFO on a 23 nucleotide antiparallel duplex was choosen to ensure the outmost stability of the underlying duplex. Unlabelled; FAM/Cy5 and FAM/BHQ1 labelled probes were purchased from TAG Copenhagen A/S (Copenhagen, Denmark) on a 0.04 μmol synthesis scale with High Performance Liquid Chromatography (HPLC) purification and Mass Spectrometry control. ATTO495/ATTO647N labelled probes were purchased from ATTO-Tec GmbH (Siegen, Germany) on a 0.2 μmol synthesis scale with HPLC purification (Figure 1b, c). The ATTO495 probe was synthesised as an oligonucleotide with a 3'-amino-modifier-C7 and linked to an ATTO495 NHS-ester, whereas the ATTO647N probe was synthesised as an oligonucleotide with a 5'-amino-modifier-C6 and linked to the ATTO647N NHS-ester. ATTO495 is derived from acridine orange, whereas ATTO647N is patent dependent. According to ATTO-Tec the ATTO647N is a mixture of two isomers with a net cationic charge of one after coupling.

### General LightCycler setup

All experiments were performed in 20 μl LightCycler capillaries using 10 μl of 2× concentration buffer with oligonucleotides and sterile water. Probe concentrations were 0.5 μM of each oligonucleotide for antiparallel duplex formation, and 1.0 μM of each oligonucleotide for parallel triplex formation. The capillaries were centrifuged and run on a LightCycler 2.0 (Roche Applied Science, Basel, Switzerland) using our standard program (Figure 5b). The standard LightCycler program consists of 1) a dissociation step from 37 to 95°C with a ramp rate of 0.2°C per second and hold for 15 seconds at 95°C, 2) annealing from 95 to 37°C with a ramp rate of 0.2°C per second and hold for 5 minutes at 37°C and 3) the dissociation step from 37 to 95°C with a ramp rate of 0.05°C per second and continued measurement of fluorescence (Figure 5b). Tm was identified using the LightCycler Software 4.1 for melting curve analysis and defined as the peak of the negative first derivative (-dF/dT).

### Buffers

All four fluorophores were initially evaluated in a sodium cacodylate buffer with a final concentration of 20 mM sodium cacodylate with 100 mM sodium chloride and 10 mM magnesium chloride. All chemicals were purchased from Sigma-Aldrich Inc (St. Louis, MO, USA). Buffers were prepared from pH 4.5 to 7.5 in 0.25 pH increments and checked with a portable pH meter (PHM 201, Radiometer Analytical, Brønshøj, Denmark). Buffers were calibrated with 1 mM sodium hydroxid and/or 1 mM hydrogen chloride. Due to the toxicity of sodium cacodylate buffer, the buffer system was changed to a sodium acetate buffer for parallel triplex formation and a sodium phosphate buffer for antiparallel duplex formation. The sodium acetate buffer had a final concentration of 50 mM sodium acetate with 100 mM sodium chloride and 10 mM magnesium chloride and the sodium phosphate buffer had a final concentration of 50 mM sodium phosphate with 100 mM sodium chloride and 0.1 mM EDTA. Sodium acetate buffers were made in the pH range from 4.5 to 6.5 with 0.25 pH steps and sodium phosphate buffers were made in the pH range from 5.5 to 8 with 0.5 pH steps.

### Evaluation of FRET and quencher pairs

All four fluorophores were investigated in all sodium cacodylate buffers. For antiparallel duplex formation, both FRET pairs were run in the sodium cacodylate and sodium phosphate buffers, whereas all parallel triplex formation FRET pairs were run in the sodium cacodylate and sodium acetate buffers. Melting curve determination for pH dependence was conducted as single measurements.

### Background Compensation

To avoid cross-talk between the LightCycler channels colour compensation for antiparallel duplex formation of each fluorophore in sodium phosphate and for parallel triplex formation of each fluorophore in sodium acetate buffer was conducted. The colour compensations were performed according to the LightCycler 2.0 Software 4.1 manual and used in all further experiments.

### LightCycler program validation

For LightCycler program validation the antiparallel duplex probes were measured at pH 6, 7 and 8 and the parallel triplex probes were measured at pH 5, 5.25, 5.5 and 5.75. The melting point was identified as the mean of four measurements at each pH. All 16 LightCycler runs were conducted within two days using the same 28 capillaries. To ensure that multiple runs did not influence the melting point determination, the standard program was run twice as the first and last run (Figure 5).

### General validation

All validation experiments were conducted using our LightCycler standard program with standard probe concentrations. The intra-assay variation for antiparallel duplex formation was determined in sodium phosphate buffer, pH 7, using 12 independent capillaries and the inter-assay variation was determined by running of 12 independent capillaries per day for six days. The inter-machine variation was determined by running 12 capillaries made from a single master mix on two LightCycler 2.0. The stability of mixed probes kept at 4°C and used for melting point determination was evaluated using 36 independent capillaries and running six new capillaries after 0, 4, 24, 48, 120 and 192 hours. Evaluation of fluorophore stability over time and after multiple runs was conducted using 24 independent capillaries leaving 12 capillaries at 4°C and 12 capillaries at room temperature and rerunning all capillaries after four hours, 1, 2, 5, 8, 20, 28, 57 and 141 days.

The validation for parallel triplex formation was conducted in sodium acetate buffer, pH 4.8, using the same setup as described for antiparallel duplex formation. As the only difference, the capillaries for the evaluation of fluorophore stability were rerun after 4 hours, 1, 2, 4, 8, 15, 22, 57 and 177 days.

### UV-absorbance and LightCycler measurements

Melting curve determinations on the LightCycler were run in quadruplicate using sodium phosphate buffer, pH 7, for antiparallel duplex and sodium acetate buffer, pH 5, for parallel triplex formation and standard probe concentrations. The same probe and buffer combinations were run on a PerkinElmer UV-vis spectrometer, Lambda 35, fitted with PTP-6 temperature programmer with five cuvettes and a temperature control cuvette. Each experiment was conducted in quadruplicate using 1 μM of each probe in 1 mL cuvettes with 2× buffer and sterile water. Melting measurements were conducted by heating to 85°C for 10 minutes followed by cooling to 8°C for 30 minutes and dissociation measurements from 10°C to 85°C as 1°C/minute. Melting measurements were recorded at 260 nm.

### Statistics

All statistics were conducted using SAS 9.1 (SAS Institute Inc, Cary, NC, USA) with the level of significance of 0.05. Intra- and inter-assay variations were determined as standard deviation divided by average mean multiplied by 100%. Inter-machine variation was determined by two-sample paired T-test of means. The samples were prepared individually, the assumption of same variance was checked by Bland-Altman plot of difference towards average, and normal distribution was checked by probability plot. The stability of mixed probes was investigated using one-way analysis of variance with Bonferroni correction for multiple tests. Each sample was prepared individually and the assumption of same variance for each group was checked for residuals as a function of predicted values. Likewise, the normal distribution assumption was checked by probability plot of the residuals. The variance of Tm determinations was evaluated by two-sample test of variances. Samples were prepared individually and normal distributions were checked by probability plot. All experiments fitted the model requirements. Percentual decrease/increase in fluorescence for antiparallel duplex formations and FAM-Cy5 parallel triplex FRET, pH 4.75 was calculated as the difference from 60°C to 85°C and for ATTO and FAM-BHQ1 parallel triplex, pH 5.75 as the difference from 37°C to 50°C.

## Competing interests

The authors declare that they have no competing interests.

## Authors' contributions

UVS and JKS did the experimental work on the LightCycler. IG and EBP did the UV absorbance measurements. UVS and GL designed the setup, planned the experiments and performed the data analysis with substantial input from the other authors. UVS and GL prepared the manuscript and all authors contributed to editing the manuscript. All authors approved the final manuscript.
